# Rationally Designed Pentapeptide Analogs of Aβ19–23 Fragment as Potent Inhibitors of Aβ42 Aggregation

**DOI:** 10.3390/molecules30092071

**Published:** 2025-05-07

**Authors:** Sachin B. Baravkar, Yan Lu, Qi Zhao, Hongying Peng, Weilie Zhou, Song Hong

**Affiliations:** 1 Neuroscience Center of Excellence, School of Medicine, Louisiana State University Health, New Orleans, LA 70112, USA; sbaravkar150@gmail.com (S.B.B.);; 2 NMR Laboratory, Department of Chemistry, Tulane University, New Orleans, LA 70115, USA; qzhao@tulane.edu; 3 Department of Environmental Health, University of Cincinnati College of Medicine, Cincinnati, OH 45221, USA; 4 Department of Physics & Adavanced Materials Research Institute (AMRI), University of New Orleans, New Orleans, LA 70148, USA; 5 Department of Ophthalmology, School of Medicine, Louisiana State University Health, New Orleans, LA 70112, USA

**Keywords:** Alzheimer’s disease, amyloid beta aggregation inhibitor, pentapeptides, circular dichroism, thioflavin T fluorescence assay, transmission electron microscopy TEM, HSQC NMR

## Abstract

Amyloid beta (Aβ42 and Aβ40) aggregation, along with neurofibrillary tangles, is one of the major neurotoxic events responsible for the onset of Alzheimer’s disease. Many potent peptide-based inhibitors mainly focusing on central hydrophobic core Aβ16–20 (KLVFF) have been reported in recent years. Herein, we report pentapeptides **1**–**4**, based on the β-turn-inducing fragment Aβ19–23 (FFAED). The synthesis of peptides **1**–**4** was carried out using Fmoc/tBu-based solid-phase peptide synthesis technique, and it was found that pentapeptide **3** potently inhibit the aggregation propensity of Aβ42, when incubated with it at 37 °C for 48 h. The aggregation inhibition study was conducted using thioflavin T-based fluorescence assay and circular dichroism spectroscopy, and supported by transmission electron microscope imaging. The conformational change on the aggregation of Aβ42 and aggregation inhibition by peptides **1**–**4** was further evaluated using ^1^H–^15^N HSQC NMR spectroscopy. The results demonstrated that the most potent analog, peptide **3**, effectively disrupts the aggregation process. This study is the first to demonstrate that an Aβ19–23 fragment mimic can disrupt the aggregation propensity of Aβ42.

## 1. Introduction

Alzheimer’s disease (AD), one of the most common dementia-related diseases (60–80% of dementia cases), is a degenerative disease generally characterized by the destruction of nerve cells and the breaking of the neural connections that enable cognition, leading to memory impairment and negatively impacting patients’ daily lives [1]. Globally, 55 million people currently suffer from dementia, with the occurrence of 10 million new cases every year, and 131.5 million people are expected to be diagnosed with it by 2050 [2]. The treatment cost of dementia-related diseases was estimated to be around USD 1.3 trillion in 2019 and is expected to increase exponentially in the future, with a new case of AD arising every 33 s; hence, in the last few decades, much effort has been expended to find a cure for this fatal disease. However, the most recent therapies revolve around symptomatic relief rather than the prevention of AD [3]. The exact cause of AD remains unknown; the major pathological characteristics include neurofibrillary tangles and amyloid plaques in the brain [4]. Extracellular amyloid plaques (also known as amyloid aggregates or simply amyloids) are solid, dense, and insoluble aggregates formed by the misfolding of the amyloid beta (Aβ) peptide, which is produced by neurons [5]. The Aβ peptide, a small (39–43 amino acids), truncated peptide, is obtained after endo-proteolytic cleavage of the precursor protein, amyloid precursor protein (APP), by γ-secretase and β-secretase enzymes [6,7]. There are two main variants of amyloid peptides, the 40-residue peptide (Aβ40) and 42-residue isoform (Aβ42), which differ only by 2 amino acids. Among these variants, the Aβ40 is the most abundant product of cleavage, followed by the Aβ42 [8]. However, the amyloid aggregates from Aβ42 are found to be more neurotoxic in nature, and hence, Aβ42 is the most studied target in AD plaque therapies [9].

The monomeric form of Aβ is a non-aggregated metabolic product which adopts a random coil conformation in aqueous solution, but with a possibility to show other secondary structures especially in AD patients [10,11,12]. The aggregation process of Aβ is not yet fully understood, but there are three key regions important for the nucleation of β-sheet-rich secondary structures. These are the N-terminal hydrophilic domain (His13-Lys16) [13,14] the central hydrophobic cluster (CHC; Leu17-Ala21, LVFFA) [15], and the highly hydrophobic C-terminal region (Ile32-Val40/Ala42), [16]. It is believed that the aggregation is often primarily driven by the two aromatic Phenylalanine residues of the CHC [17,18]. Hence, most therapeutic strategies involved are to target neurotoxic Aβ aggregates, which can be accomplished by maintaining Aβ in its non-toxic monomeric form, disintegrating oligomers, or remodeling the oligomers into nontoxic off-pathway aggregates [19]. With this approach in mind, a variety of small molecules, peptides and peptidomimetics have been identified as inhibitors of amyloid plaques [20,21,22]. Due to their non-selectivity and low binding affinity towards AB42 peptide, current small molecule inhibitors are not ideal, with most of these compounds having failed in clinical trials due to low efficacy and serious side effects [23]. On the other hand, peptide-based strategies have shown promising results due to their strong binding propensity to the protein and high synthetic feasibility for structural modification [24]. A variety of peptide-based inhibitors, including synthetic peptides [24,25,26,27,28,29,30,31,32,33,34,35,36], antimicrobial peptides [37,38,39,40], cell-permeable peptides [41], peptide foldamers [21,42] and peptide polymer conjugates [43] have been developed specifically to reduce the aggregation-propensity of Aβ42.

**Scheme 1:**
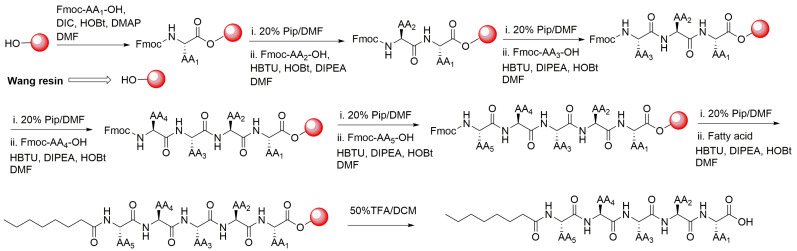
General strategy for Fmoc/tBu-based solid-phase peptide synthesis (SPPS). Wang resin was used to obtain C-terminal carboxyl.

Short peptide sequences derived from natural Aβ-sequence were able to bind to Aβ owing to the identification of the CHC and its key residues constituents. KLVFF was the first short peptide inhibitor of amyloid aggregation. It was followed by a truncated analog, QKLVFF, which was able to bind Aβ42 and inhibit its assembly into fibrils [44,45]. Over the past two decades, many short peptides targeting CHC region KLVFF have been developed as inhibitors against Aβ42 and Aβ40 [46]. Some of the important structural features of these peptide inhibitors include β-sheet breaker peptides such as Ac-LPFFD-NH_2_, where the proline acts as β-sheet breaker due to its rigid five membered core [47]. Other active analogs include Ac-LPFFN-NH_2_ and LPYFD [48,49]. Further improvements in the design of peptide inhibitors comprise the use of unnatural amino acids [such as D-amino acids, α-aminoisobutyric acid (Aib), dehydrophenylalanine (ΔPhe), and halogenated Phe) [50,51,52,53], peptide-bioactive small molecule conjugates (such as Trehalose, Cholyl, Fc) [54,55,56], and macrocyclic peptides [57,58]. One of the recently developed stable, non-cytotoxic ααα and ααγ-hybrid pentadecapeptide showed that the inhibition of aggregation of Aβ42 and reduces the toxicity of Aβ42 [59,60]. Another hybrid octapeptide containing an anthranilic acid unit (Ant, 2-aminobenzoic acid) as a β-sheet breaker not only inhibited Aβ42 amyloid formation but also disrupted the mature Aβ42 fibrils into nontoxic, small-molecular-weight, soluble species [61]. Inspired by the immense potential of short peptides as inhibitors of amyloid beta, we developed pentapeptides which resemble with the previously unexplored central hydrophobic region FFAED (Aβ19–23). This sequence contains two negatively charged amino acids, which can help increase the hydrophilicity as well as improve the intermolecular interactions via hydrogen bonding. Phenylalanine at N-terminus helps balance the hydrophobic and hydrophilic properties. Our hypothesis was that our synthesized pentapeptides (**1**–**4**, shown in Scheme 1) would bind to Aβ42, prevent its aggregation, and consequently reduce brain neurotoxicity. The pentapeptides were synthesized using standard Fmoc/tBu based solid-phase synthesis techniques. Their aggregation inhibition studies were conducted using thioflavin T fluorescence measurements and transmission electron microscopic images. Conformational changes in the secondary structure of Aβ42 upon binding with pentapeptides were observed using a circular dichroism (CD) spectroscopy and Heteronuclear Single Quantum Coherence Nuclear Magnetic Resonance spectroscopy (HSQC NMR). HSQC NMR is a two-dimensional NMR technique used to correlate protons (^1^H) with heteronuclei such as carbon-13 (^13^C) or nitrogen-15 (^15^N) [62].

## 2. Result and Discussion

### 2.1. Peptide Design and Synthesis

**Figure 1:**
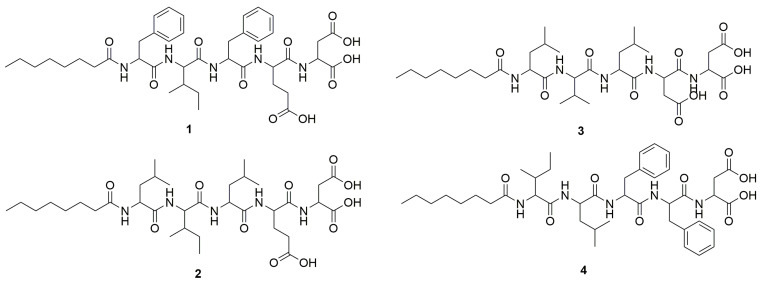
Chemical structure of peptides **1**–**4**.

The design of the peptide-based inhibitors was focused on the central hydrophobic region of the Aβ42 peptide (residues 16–20) and β-turn-inducing region (residues 22–27) [63]. As discussed previously, many inhibitors based on the central hydrophobic region (residues 16–22) have been reported [64]. However, there are no inhibitors targeting the β-turn-forming sequence FFAED (Aβ19–23). We rationally designed four diverse pentapeptide analogs of the FFAED sequence (Aβ19–23) where we included β-sheet-breaking amino acids such as Leu, Ile, and Val and we hypothesized that these peptides would inhibit β-turn-inducing properties shown by FFAED (as shown in Figure 1). Based on our previous experience, Phe (F) tends to form aggregates due to π-π stacking between the molecules. The first sequence designed was pentapeptide 1, C8-FIFED, where we changed the positions of two Phe (F) with the inclusion of Ile (I) in between them. The rationale behind separating two Phe derivatives was to reduce the hydrophobicity and π-π stacking due to two adjacent Phe analogs; also, the inclusion of β-sheet-breaking amino acid Ile in between two Phe would be detrimental for β-sheet formation. The free amine group at the N-terminus was acylated with a short fatty acid (caprylic acid C8) to avoid inter- and intra-molecular H-bonding interactions and the C-terminus amino acids (Glu E and Asp D) were unchanged to exert similar hydrophilic interactions. Further, we replaced Phe with β-sheet-breaking aliphatic amino acids Leucine (L) and Valine (V) to generate pentapeptide 2, C8-LILED, and pentapeptide 3, C8-LVLDD. We exchanged Glu with Asp in peptide 3 to understand any changes due to shortening of the side chain.

On the other hand, pentapeptide 4, C8-ILFFD, was generated by removing one of the negatively charged polar amino acids (E or D) with Phe. Peptide **4** containing adjacent Phe derivatives was expected to exert similar properties as parent FFAED truncated peptide and hence can be used as a control for the bioassays. The synthesis of peptides **1**–**4** was carried out using Fmoc/tBu-based solid-phase peptide synthesis on Wang resin as shown in Scheme 1. The obtained peptides were purified, lyophilized, and confirmed for their structures using NMR and liquid chromatography collision-induced dissociation tandem mass spectrometry (LC-CID-MS/MS).

### 2.2. The Inhibition of Aβ42 Aggregation by Pentapeptides Using Thioflavin T (ThT) Fluorescence-Based Kinetic Assay

**Figure 2:**
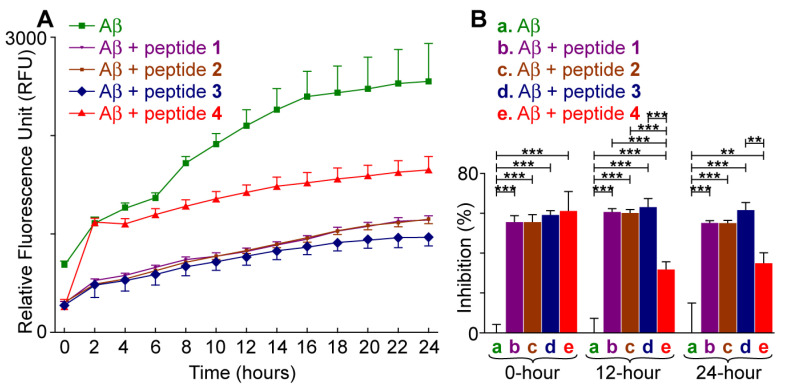
Specific pentapeptide analogs of Aβ19–23 fragment inhibited Aβ42 aggregation in ThT fluorescence-based Aβ aggregation assay. (**A**) Time-dependent ThT fluorescence-based aggregation study of Aβ42 (20 µM) with peptide **1**, **2**, **3**, or **4** of pentapeptide analogs of Aβ19–23 fragment. Aβ42/peptide (100 µM) in phosphate buffer solution (10 mM, pH 7.4, 37 C). (**B**) Inhibition of Aβ42 aggregation fluorescence (%) relative to aggregation of Aβ42 alone by each peptide at 0 h, 12 h, or 24 h of incubation. Each data point is mean of data acquired from three independent experiments. Error bars show standard deviations. ** *p* < 0.01, *** *p* < 0.001.

ThT is a small molecule fluorescent dye that shows enhanced fluorescence intensity when it binds to amyloid aggregates. The aggregation kinetics of the Aβ42 peptide was studied using a ThT fluorescence assay [65]. Aβ42 peptide (20 µM) was incubated at 37 °C with or without pentapeptide **1**, **2**, **3**, or **4** (100 µM). The ratio of Aβ42/pentapeptide was maintained at 1:5. In the absence of any pentapeptides **1**–**4**, Aβ42 showed a steady increase in the fluorescence signal along with the increase in incubation time (Figure 2A). The fluorescence signal reached a threshold and saturated after the complete aggregation of Aβ42 peptide that resulted in a turbid solution. We observed remarkable inhibition of Aβ42 by all four peptides upon incubation with Aβ42. We observed significant inhibition of Aβ42 by all four peptides, with inhibition ranging from 55% to 61% upon initial incubation with Aβ42. At 12 h, the inhibition slightly increased for peptides **1**–**3** (60–64%) but decreased for peptide **4** (32.2%). By the 24 h point, peptide **3** maintained strong inhibitory activity, while the effectiveness of peptides **1** and **2** slightly declined. Among this series of peptides, pentapeptide **3** showed the best results and was then taken as a lead inhibitor for further analysis of its ability to inhibit aggregate formation.

### 2.3. Secondary Structural Changes in Aβ42 Using CD Spectroscopy

**Figure 3:**
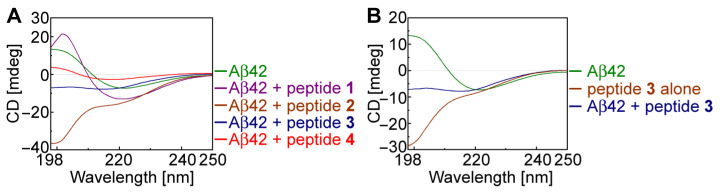
Secondary structural investigation of Aβ42 with and without pentapeptides using CD spectroscopy. (**A**) CD spectra of Aβ42 (40 µM, green line), Aβ42/peptide **1** (40:200 µM, purple line), Aβ42/peptide **2** (40:200 µM, brown line), Aβ42/peptide **3** (40:200 µM, dark blue line), and Aβ42/peptide **4** (40:200 µM, red line). (**B**) CD spectra of Aβ42 alone (green line), Aβ42/peptide **3** (dark blue line), and peptide **3** alone (brown line). CD experiments were carried out in phosphate buffer solution (10 mM, pH 7.4, 37 °C). Scan wavelength range was 198–250 nm.

Aβ42 peptide in its monomeric state adopts a random coil conformation with no significant signals but slowly changes its conformation to β-sheet during its aggregation [66]. To understand the conformational changes in Aβ42, we conducted CD spectroscopic studies with and without pentapeptides in a phosphate buffer solution at 37 C. The concentrations of Aβ42 and an inhibitor peptide were maintained at a ratio of 1:5 (40:200 µM). When the Aβ42 was incubated alone for 48 h, we observed that it exhibited typical β-sheet conformation with a negative minimum signal at 220 nm and a positive signal around 198–200 nm (Figure 3A,B, green line). The CD spectra of Aβ42 incubated with peptides **2** (Figure 3A, brown line) and **3** (Figure 3A, dark blue line) showed random coil conformation, which suggested that peptides **2** and **3** disrupted the β-sheet conformation of Aβ42. When we compared the CD spectra of the most potent peptide **3** alone (Figure 3B, brown line) with the Aβ42/peptide **3** sample (Figure 3B, blue line), we observed that both adopted random coil conformation, and peptide **3** disrupted the β-sheet conformation of Aβ42.

### 2.4. Investigation of Aβ42 Aggregation and Its Inhibition Using Transmission Electron Microscopy (TEM) Analysis

**Figure 4:**
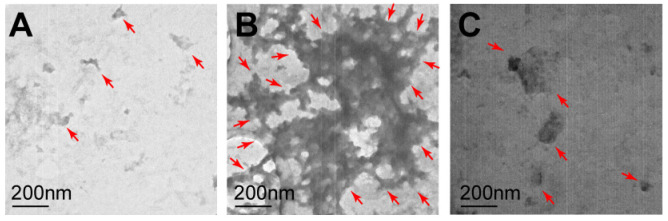
TEM analysis of Aβ42 with and without inhibitor peptide 3. (**A**) Aβ42 alone at 0 h; (**B**) Aβ42 was incubated without peptide 3 at 37 °C for 48 h; (**C**) Aβ42 was incubated with peptide 3 at 37 °C for 48 h. Red arrows indicate aggregate deposits. Scale bar was 200 nm.

To observe the aggregation propensity of Aβ42, we undertook its morphological imaging via TEM analysis. TEM analysis of Aβ42 in the absence and presence of peptide **3** was carried out using reported methods [67]. The concentration of Aβ42 and peptide **3** was maintained in the ratio 1:5 (10:50 µM). There were initially no aggregate deposits of Aβ42 (at time 0 h, Figure 4A) but we observed large fibrillar aggregate deposits when Aβ42 was incubated at 37 °C for 48 h (Figure 4B). When Aβ42 was incubated with peptide **3** at 37 °C for 48 h, we no longer found any aggregated network of fibrils (Figure 4C) since peptide **3** diminished the aggregation propensity of Aβ42.

### 2.5. ^1^H–^15^N HSQC NMR Revealed Conformational Changes of Aβ42 by Peptide 3

**Figure 5:**
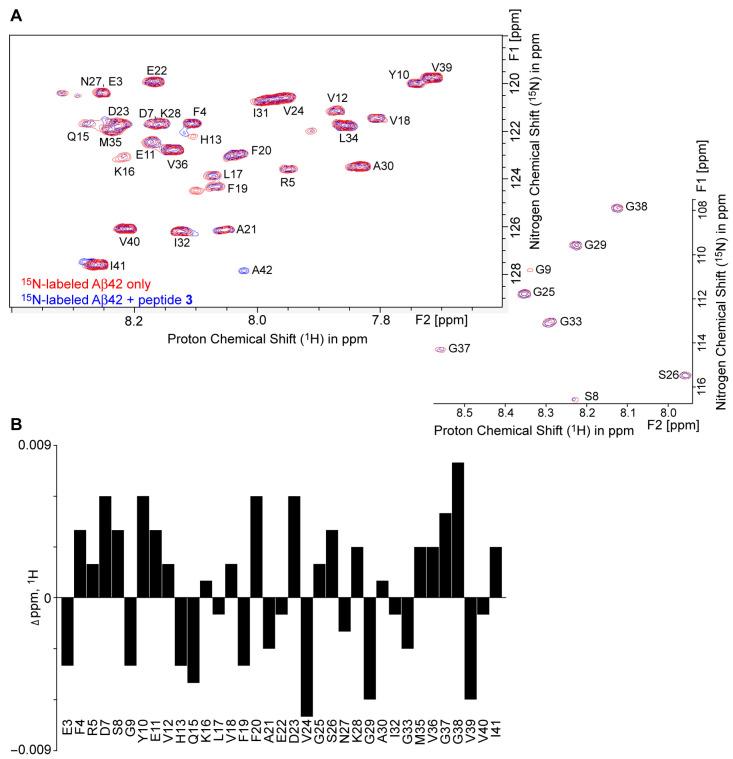
The ^1^H–^15^N HSQC NMR experimental results of ^15^N-labeled Aβ42 with and without inhibitor peptide 3. ^1^H–^15^N HSQC NMR spectral data of ^15^N-labeled Aβ42, i.e., ^15^N-Aβ42, with and without peptide **3** were acquired in 9:1 H_2_O/D_2_O with 20 mM sodium phosphate buffer (pH 7.4) and overlaid. (**A**) The representative ^1^H–^15^N HSQC NMR spectral contour plot. (**B**) **^1^**H chemical shifts were increased with positive perturbations (Δs, ppm) or decreased with negative Δs after the addition of peptide **3** in the Aβ42 solution **(**^15^N-Aβ42/peptide **3** = 1:1.1 concentration ratio). The peaks are labeled according to the assignments established in references [68,69].

The conformational changes in the secondary structure of Aβ42 when incubated with pentapeptides can be understood from ^1^H–^15^N HSQC NMR studies. When the rationally designed peptide binds to native ^15^N-labeled Aβ42 (^15^N-Aβ42), there is a change in the chemical shift values and peak intensities of amide protons [22,31,59,60]. The examination of overlay HSQC spectra of ^15^N-Aβ42 with and without peptide **3** revealed a small perturbation in the amide NH chemical shifts of ^15^N-Aβ42 in the presence of peptide **3** (^15^N-Aβ42/peptide **3** = 1:1.1 concentration ratio) (Figure 5A), where the spectral data were displayed using the same contour levels, with identical sample concentration and acquisition parameters. Mostly, the residues D7, Y10, Q15, F20, D23, V24, G29, G37, G38, and V39 (Figure 5B) showed a measurable change in their ^1^H chemical shifts, while F4, G9, K16, F19, G37, G38, and I41 (Table S1) displayed change in their ^15^N chemical shifts after the addition of peptide **3**. We also noticed the change in the peak intensities of ^15^N-Aβ42 residues, including Y10, V12, V18, F20, E22, V24, I31, and V39, upon interaction with peptide **3** from the contour levels of the cross peaks (Table S2). In addition, ^15^N-A42 only showed in the spectrum of the sample of ^15^N-Aβ42 + peptide **3** under the current acquisition parameter. These changes in the HSQC spectrum of ^15^N-Aβ42 in the presence of peptide **3**, which can be attributed to the interaction between ^1^H–^15^N of ^15^N-Aβ42. The analysis of HSQC experiments suggests that the peptide **3** can affect the conformation in ^15^N-Aβ42 after interacting at different amino acid residues and C terminus. The results of the ^1^H–^15^N HSQC NMR analysis support the results observed in the fluorescence assay and the TEM and circular dichroism experiments. The more quantitative features, kinetics, dose-responses, and temperature-dependence of peptide-3 disturbance of ^1^H and^15^N interaction of ^15^N-Aβ42 should be studied in the future with comprehensive and systematic NMR approaches.

## 3. Materials and Methods

### 3.1. Materials

Fmoc-protected amino acids—such as Fmoc-Val-OH (CAS No. 68858-20-8, 99.56%), Fmoc-Leu-OH (CAS No. 35661-60-0, 99.78%), Fmoc-Ile-OH (CAS No. 71989-23-6, 98%), Fmoc-Phe-OH (CAS No. 35661-40-6, 99.92%), Fmoc-Asp(OtBu)-OH (CAS No. 71989-14-5, 99.92%), Fmoc-Glu(OtBu)-OH (CAS No. 71989-18-9, 99.92%)—and fatty acids, namely n-octanoic acid (CAS No. 124-07-2, 99.56%), were obtained from BLD Pharmatech Co., Ltd., Cincinnati, OH, USA, and were used without further purification. Wang resin (0.9 mmol/g, 100–200 mesh), the Kaiser test kit (Catalog no. KGZ001), and O-Benzotriazole-N, N, N′, N′-tetramethyluronium-hexafluoro-phosphate (CAS No. 94790-37-1, 1-) were obtained from Aapptec, LLC, Louisville, KY, USA. Hydroxybenzotriazole (HOBt, CAS No. 2592-95-2, 98.75%) was purchased from Apexbio, Houston, TX, USA. Trifluoroacetic acid (CAS No. 76-05-1, 99%) was obtained from Honeywell Research Chemicals, Muskegon, MI, USA. Piperidine (CAS No. 110-89-4, 99%) was acquired from Sigma-Aldrich Co., LLC, St. Louis, MO, USA. N, N-diisopropylethylamine (CAS No. 7087-68-5, 99%) was purchased from TCI America, Portland, OR, USA. N, N-dimethylformamide (CAS No. 68-12-2), diethyl ether (CAS No. 60-29-7), dichloromethane (DCM, CAS No. 75-09-2), N, N′-Diisopropylcarbodiimide (DIC, CAS No. 693-13-0) and 4-(Dimethylamino)pyridine (DMAP, CAS No. 100-10-7) were sourced from Thermo-Scientific, Ward Hill, MA, USA.

### 3.2. Solid-Phase Synthesis of Peptides (SPPS)

The fatty acid-conjugated peptides were manually synthesized. We used Fmoc/tBu-based solid-phase peptide synthesis for this process. Poly-Prep columns sourced from Bio-Rad Laboratories (Hercules, CA, USA) were utilized. The synthesis was performed on a 0.1 mmol scale on Wang resin, as reported previously [70,71].

#### 3.2.1. General Procedure for SPPS of Peptides **1–4** (Example of SPPS of Peptide **3**)

The Wang resin (111 mg) was swollen in 5.0 mL DCM in a Bio-Rad column (Hercules, CA, USA) for 30 min. Positive pressure was applied to expel the solvent. The Fmoc-Asp(tBu)-OH (164 mg, 4 equiv.) was placed in a scintillation vial and dissolved in 5.0 mL DMF along with DIC (50 mg), HOBt (54 mg), and DMAP (5.0 mg). The mixture was sonicated for 1 min before being transferred to a resin column. The column was then shaken on a vortex mixer for 6 h. The solvent was removed from the column, which was then washed with DMF (3 × 5 mL) and DCM (3 × 5 mL). The resin was end-capped by adding 5 mL acetic anhydride: pyridine solution (3:2, *v*/*v*) and rocking the resin for 1 h. This was followed by washing with DMF (3 × 5 mL) and DCM (3 × 5 mL). The Fmoc group was removed by adding 20% piperidine in DMF (5.0 mL) and shaking for 20 min. The solvent was removed, and the column was washed with DMF (3 × 5 mL) and DCM (3 × 5 mL), which was confirmed by a positive Kaiser test.

In a 10 mL scintillation vial, the second amino acid (4 equiv), HBTU (152 mg), HOBt (54 mg), and DIPEA (0.2 mL) were dissolved in 5.0 mL DMF. This mixture was thoroughly shaken for 10 min before adding to the column, which was then shaken on a vortex mixer for 6 h. Solvent was removed from the column, which was then washed with DMF (3 × 5 mL) and DCM (3 × 5 mL) (confirmed by a negative Kaiser test). Deprotection was performed using 20% piperidine in 5.0 mL DMF.

The coupling procedure was repeated for coupling other amino acids and a fatty acid. To cleave the fatty acid–peptide conjugate from resin, a mixture of 50% TFA/DCM (5.0 mL) was added, and the mixture was stirred at room temperature for 2 h. The solution containing TFA was then filtered from the column, and the filtrate was evaporated using a rotary evaporator to remove excess TFA.

Ice-cold diethyl ether (25 mL) was added to the resulting crude product, forming a white, solid precipitate. The mixture was centrifuged for 5 min, the ether was removed by decantation, and the precipitated compound was washed three times with diethyl ether. The resulting compound was dried using a rotary evaporator, then lyophilized for 24 h in a freeze-dryer (Thermo Savant, Holbrook, NY, USA). Its structure was confirmed by LC-MS/MS and NMR analysis.

#### 3.2.2. Procedures to Remove Trifluoracetic Acid Counter-Ions from Fatty Acid–Peptide Conjugates by Counter-Anion Exchange

The trifluoroacetic acid counter-anion was replaced with HCl. This was achieved by dissolving the white precipitate in 0.1 M HCl solution (5.0 mL). The mixture was stirred for 15 min. Then, 5 mL acetonitrile was added. The resulting soluble mixture was dried in a dry-ice bath and lyophilized overnight to yield the HCl salt of the peptide.

#### 3.2.3. Determination of the Molecular Structures and Quantities of Compounds

The reagents and the fatty acid–amino acid/peptide conjugates were analyzed using an LC-CID tandem MS/MS system. This system consisted of an Agilent 1100 LC system (HPLC-DAD-autosampler, Agilent Technologies, Inc., Santa Clara, CA, USA) and a QTRAP 6500^+^ quadruple-linear trap tandem mass spectrophotometer with electrospray ionization (Sciex.com, Framingham, MA, USA).

The NMR analysis was carried out using a Brucker 400 MHz NMR instrument. For this, DMSO-d_6_ (CAS No. 2206-27-1, 99.9 atom% D, Thermo-Scientific, Fair Lawn, NJ, USA) was used as a solvent. The analysis utilized Topspin 4.3.0 version software (Bruker Corporation 40 Manning Road Billerica, MA, USA). The ^1^H NMR (400 or 500 MHz), ^13^C NMR (100.5 or 125.8 MHz) and ^13^C DEPT data were obtained from a purified compound (20 mg) dissolved in 0.7 mL DMSO-d_6_ in a 5 mm diameter NMR tube. DEPT-135 was used to determine the multiplicity of carbon atoms. In this process, CH_2_ groups showed inverted signals while CH and CH_3_ groups appeared upright. Quaternary carbon (C) did not show any signal.

The designed molecular structures of compounds **1**–**4** were verified by their NMR spectra (Figures S1–S10 in Supplemental Materials) that provided the following chemical shifts δ and coupling constants J, and by CID-MS/MS analysis that provided their molecular masses and structure-dependent fragment ions below as we did previously for C8-tripeptide conjugates [71,72].

Peptide **1** [C8-FIFED]: ^1^H NMR (400 MHz, DMSO-d_6_) δ 12.40 (b, 2 H), 7.67–8.33 (m, 5 H), 7.09–7.31 (m, 10 H), 4.06–4.70 (m, 5 H), 2.59–3.12 (m, 6 H), 2.17–2.35 (m, 2 H), 1.88–2.08 (m, 3 H), 1.59–1.85 (m, 2 H), 1.10–1.45 (m, 12 H), and 0.89–0.69 (m, 9 H); ^13^C NMR (100.5 MHz, DMSO-d_6_) δ 174.17, 173.43, 172.62, 171.67, 171.56, 171.45, 171.25, 171.00, 138.55, 138.53, 138.07, 137.90, 129.52, 129.43, 128.57, 128.42, 128.31, 126.60, 126.48, 57.17, 53.95, 53.79, 53.65, 51.59, 37.31, 35.56, 31.53, 30.34, 28.87, 28.75, 26.81, 25.65, 24.41, 22.47, 15.58, 14.38, and 11.46. Its CID-MS/MS fragmentation ions verified its molecular mass M and structure, including ions of *m/z* 794 [M–H^+^], 776 [794–H_2_O], 667, 663, 535, 521, 408, 373, 274, 131, and 114. The isolated quantity was 47 mg (overall yield—59%, purity—~94%).

Peptide **2** [C8-LILED]: ^1^H NMR (500 MHz, DMSO-d_6_) δ 8.13 (d, J = 8.3 Hz, 1 H), 8.00 (d, J = 8.2 Hz, 1 H), 7.78 (d, J = 9 Hz, 1 H), 7.45 (d, J = 7.0 Hz, 1 H), 4.35–4.19 (m, 2 H), 4.15 (t, J = 8.4 Hz, 1 H), 3.98 (dt, J = 8.6, 6.3 Hz, 1 H), 2.20–1.98 (m, 6 H), 1.88–1.34 (m, 13 H), 1.29–1.15 (m, 8 H), and 0.92–0.74 (m, 21 H). Its CID-MS/MS fragmentation ions verified its molecular mass M and structure, including ions of *m/z* 726 [M–H^+^], 708 [726–H_2_O], 595, 487, 466, 535, 374, 353, 240, 131, and 114. The isolated quantity was 40 mg (overall yield—62%, purity—~95%).

Peptide **3** [C8-LVLDD]: ^1^H NMR (500 MHz, DMSO-d_6_) δ 12.68 (b, 3 H), 8.12 (d, *J* = 8.1 Hz, 1 H), 7.96 (d, *J* = 8.1 Hz, 1 H), 7.96 (d, *J* = 8.1 Hz, 1 H), 7.83 (d, *J* = 6.8 Hz, 1 H), 7.70 (d, *J* = 8.8 Hz, 1 H), 4.53 (m, 1 H), 4.40 (m, 1 H), 4.31 (m, 2 H), 4.11 (m, 1 H), 4.53 (m, 1 H), 2.68–2.39 (m, 4 H), 2.09 (m, 2 H), 1.94 (m, 1 H),1.65–1.36 (m, 8 H), 1.23–1.18 (m, 8 H), and 0.91–0.75 (m, 21 H). ^13^C NMR (125.8 MHz, DMSO-d_6_) δ 172.31, 172.24, 172.15, 171.84, 171.75, 171.60, 170.62, 170.06, 57.61, 50.97, 50.75, 49.20, 48.53, 40.79, 40.49, 35.96, 35.15, 31.20, 30.43, 28.48, 28.39, 25.32, 24.20, 24.06, 23.06, 23.03, 22.07, 21.57, 21.43, 19.21, 18.16, and 13.97. Its CID-MS/MS fragmentation ions verified its molecular mass M and structure, including ions of *m/z* 698 [M–H^+^], 680 [698–H_2_O], 572, 567, 459, 438, 360, 339, 276, 131, and 114. The isolated quantity was 40 mg (overall yield—58%, purity—~98%).

Peptide **4** [C8-ILFFD]: ^1^H NMR (400 MHz, DMSO-d_6_) δ 12.74 (b, 2 H), 8.23 (d, J = 8.2 Hz), 8.02–7.73 (m, 4 H), 7.32–7.10 (m, 10 H), 4.64–4.05 (m, 5 H), 3.08–2.86 (m, 4 H), 2.80–2.70 (m, 1 H), 2.19–2.04 (m, 3 H), 1.75–1.10 (m, 16 H) and 0.90–0.65 (m, 15 H); ^13^C NMR (100.5 MHz, DMSO-d_6_) δ 173.08, 172.58,172.51, 172.15, 171.86, 171.43, 171.17, 137.89, 137.73, 129.57, 129.49, 128.60, 128.32, 126.86, 126.56, 57.09, 53.81, 53.56, 53.47, 51.32, 41.22, 37.96, 37.13, 36.49, 35.49, 31.63, 28.88, 25.84, 24.78, 24.38, 23.42, 22.48, 21.87, 15.75, 14.37, and 11.19. Its CID-MS/MS fragmentation ions verified its molecular mass M and structure, including ions of *m/z* 778 [M–H^+^], 766 [778–H_2_O], 662, 644 [662–H_2_O], 647, 500, 426, 277, 131, and 114. The isolated quantity was 43 mg (overall yield—55%, purity—~98%).

### 3.3. Preparation of Amyloid β42

The β-amyloid (1–42), human TFA salt (CAS No. 107761-42-2) was purchased from Angel Pharmatech Ltd., Shanghai, China. The Aβ42 (0.903 mg) was dissolved in 1 mL of 1,1,1,3,3,3-hexafluoroisopropanol (HFIP) in a 5 mL Eppendorf tube and then kept for occasional vortex at room temperature for 1 h to maintain in a monomeric state. HFIP was evaporated from the sample by slow flow of nitrogen gas. The sample vials containing Aβ42 were then lyophilized overnight and stored at −80 °C until further use. Before the usage, the lyophilized Aβ42 was dissolved in 1 mL PBS buffer (1X) to make the 0.2 mM stock solutions of monomeric Aβ42 in PBS.

### 3.4. Thioflavin T (ThT) Fluorescence-Based Kinetic Assay

The aggregation kinetics of Aβ42 were studied using a ThT assay. The measurements of ThT-based fluorescence were carried out on flat-bottom, clear 96-well plates. The samples were excited at 440 nm, and the fluorescence emission was monitored at 480 nm. The stock solution of Aβ42 (0.2 mM) was prepared in PBS and incubated at 37 °C overnight. The concentration of Aβ42 was kept constant at 20 μM in all experiments. The stock solution of ThT (1 mM) in PBS (pH 7.4) was prepared by dissolving 3.19 mg ThT in 10 mL PBS. The control assay was performed by the addition of Aβ42 (20 μM) to the ThT (20 μM) in PBS, and the volume was adjusted to 200 μL. For the inhibition kinetics assay, peptides **1**–**4** were dissolved in DMSO at a 2 mM concentration and diluted in PBS as per required concentration (200 μM). The fibrillation of the Aβ42 with the appropriate concentrations of peptides **1**–**4** was studied in sodium phosphate buffer (pH 7.4), and the final volume was maintained at 200 μL. The solution containing Aβ42, ThT, and peptides was incubated for about 24 h at 37 °C and subjected to fluorescence measurements with orbital shaking for 120 s before taking each reading. Measurements were performed on SpectraMax M5 (Molecular Devices LLC, Sunnyvale, CA, USA) fluorescence plate reader in three independent experiments.

### 3.5. Circular Dichroism Spectroscopy

Circular dichroism spectroscopy analysis was carried out using a J-1100 spectrometer (Jasco Incorporated, Easton, MD, USA). For CD experiments, the stock solution of Aβ42 (200 μM) was further diluted with PBS buffer to the final concentration of (40 μM) in 300 μL. The PBS buffer was used as a blank. The 40 μM concentration Aβ42 and 200 μM of peptides were used for CD measurements. The CD spectra of Aβ42 alone were measured from 250 to 198 nm wavelength at 37 °C. The mixture containing Aβ42 and peptide (1:5 ratio) was incubated 24 h and their CD spectra were recorded at 37 °C.

### 3.6. ^1^H–^15^N HSQC NMR Spectroscopy

The experiments of ^1^H–^15^N HSQC NMR were performed using a Bruker 600 MHz NMR instrument (Bruker BioSpin, Billerica, MA, USA). The monomeric HFIP treated ^15^N-labeled Aβ42 (^15^N-Aβ42) was purchased from rPeptide Watkinsville, GA, USA. The NMR experiments were performed by maintaining the solution ratio of 9:1 (H_2_O/D_2_O) with 20 mM sodium phosphate buffer (pH 7.4). In each experiment, fresh ^15^N-Aβ42 samples (40 μM, 0.1 mg in 0.55 mL buffer) were prepared to maintain ^15^N-Aβ42 in the monomeric native form to avoid amyloid formation, and its HSQC spectrum was obtained at 283 K with a cryoprobe. A stock solution of peptide **3** (1 mM, 0.7 mg in 1 mL buffer) was prepared. The addition of peptide **3** to the ^15^N-Aβ42 sample resulted in the solution for ^1^H–^15^N HSQC NMR experiments at a concentration *ratio* 1:1.1 of ^15^N-Aβ42/peptide **3**.

### 3.7. Statistical Analysis

Statistical analysis was conducted using SAS 9.4 (Cary, NC, USA). Data were analyzed by a repeated measures analysis of variance. Pairwise comparisons between peptide groups were adjusted by Tukey’s method. A *p* ≤ 0.05 was considered statistically significant. Data are presented as mean ± standard derivation.

## 4. Conclusions

In this report, we focus on the solid-phase synthesis of pentapeptides **1**–**4**, with the goal of developing effective aggregation inhibitors for the amyloid peptide Aβ42. The structural backbone of these peptides was inspired from the β-turn inducing fragment (Aβ19–23, FFAED) of Aβ42. Peptide **3**, with the sequence C8-LVLDD-OH, showed most promising ability to inhibit the aggregation of Aβ42 owing to the presence of aliphatic, hydrophobic amino acids such as L-Leucine (L) and L-Valine (V). Similarly, the other two peptides **1** (C8-FIFED) and **2** (C8-LILED) showed comparable inhibitory activity against Aβ42 aggregation. However, peptide **4** with C8-ILFFD showed much less propensity to inhibit the aggregation. The D or E moiety is hydrophilic, with a carboxyl residue. The fourth amino acid moiety D in peptide **3** or E in peptide 1 or 2 is likely to be necessary for inhibiting Aβ42 aggregation since peptide **4** with weaker inhibition has hydrophobic F as the fourth amino acid moiety. These studies on the inhibition of Aβ42 aggregation by ultrashort pentapeptides **1**–**4** were conducted using thioflavin T fluorescence binding assay as well as CD, TEM, and ^1^H–^15^N HSQC NMR analyses. This pioneering work was our first step towards our efforts involving the synthesis and evaluation of amyloid peptide aggregation and we would like to explore further opportunities to synthesize peptide inhibitors which could possibly reverse the onset of AD rather than just provide symptomatic relief. We will test pentapeptides **1–3** in vivo using mouse and rat Alzheimer’s disease models with Aβ pathology to determine their effect on Aβ accumulation in brains and associated cognitive dysfunctions. For instance, 5xFAD [73,74,75] mouse and Tg-F344 rat models [76,77,78,79] of AD will be employed. Pentapeptides **1**–**3** can be delivered intranasally to partially bypass the blood–brain barrier [80,81,82]. This approach involves administering pentapeptides **1**–**3** in saline through the nasal passage, which facilitates more efficient delivery to the brain compared to intravenous or intra-arterial methods. Furthermore, intranasal administration is noninvasive and holds promise for practical application. Our results of these four peptides implicate the potential structure–activity relationship (SAR) of each amino acid moiety, which could be used as a new lead in the future to unravel the SAR and develop a more effective drug candidate that functions through its inhibition of Aβ42 aggregation.

## Data Availability

Data are contained within the article.

## Supplementary Materials

The following supporting information can be downloaded at: https://www.mdpi.com/article/10.3390/molecules30092071/s1, Figures S1–S10: NMR spectra of peptides 1–4. Table S1: Measurable ^15^N chemical shift changes of ^15^N-labeled Aβ42 with and without inhibitor peptide 3 in the ^1^H–^15^N HSQC NMR experiments. Table S2: Peak intensities of ^15^N-labeled Aβ42 with and without inhibitor peptide 3 in the ^1^H–^15^N HSQC NMR experiments.

## References

[1] Alzheimer’s Association (2018). 2018 Alzheimer’s disease facts and figures. Alzheimer’s Dement..

[2] World Health Organization Newsroom-Facts Sheet-Deatial-Dementia. https://www.who.int/news-room/fact-sheets/detail/dementia.

[3] DiBello J.R., Lu Y., Swartz J., Bortnichak E.A., Liaw K.L., Zhong W., Liu X. (2023). Patterns of use of symptomatic treatments for Alzheimer’s disease dementia (AD). BMC Neurol..

[4] Kasim J.K., Kavianinia I., Harris P.W.R., Brimble M.A. (2019). Three Decades of Amyloid Beta Synthesis: Challenges and Advances. Front. Chem..

[5] Yan R., Vassar R. (2014). Targeting the beta secretase BACE1 for Alzheimer’s disease therapy. Lancet Neurol..

[6] Lin Y.S., Bowman G.R., Beauchamp K.A., Pande V.S. (2012). Investigating how peptide length and a pathogenic mutation modify the structural ensemble of amyloid beta monomer. Biophys. J..

[7] Lee H.J., Korshavn K.J., Nam Y., Kang J., Paul T.J., Kerr R.A., Youn I.S., Ozbil M., Kim K.S., Ruotolo B.T. (2017). Structural and Mechanistic Insights into Development of Chemical Tools to Control Individual and Inter-Related Pathological Features in Alzheimer’s Disease. Chemistry.

[8] Selkoe D.J. (2001). Alzheimer’s disease: Genes, proteins, and therapy. Physiol. Rev..

[9] Roher A.E., Lowenson J.D., Clarke S., Wolkow C., Wang R., Cotter R.J., Reardon I.M., Zurcher-Neely H.A., Heinrikson R.L., Ball M.J. (1993). Structural alterations in the peptide backbone of beta-amyloid core protein may account for its deposition and stability in Alzheimer’s disease. J. Biol. Chem..

[10] Danielsson J., Jarvet J., Damberg P., Graslund A. (2005). The Alzheimer beta-peptide shows temperature-dependent transitions between left-handed 3-helix, beta-strand and random coil secondary structures. FEBS J..

[11] Abelein A., Abrahams J.P., Danielsson J., Graslund A., Jarvet J., Luo J., Tiiman A., Warmlander S.K. (2014). The hairpin conformation of the amyloid beta peptide is an important structural motif along the aggregation pathway. J. Biol. Inorg. Chem..

[12] Roche J., Shen Y., Lee J.H., Ying J., Bax A. (2016). Monomeric Abeta(1-40) and Abeta(1-42) Peptides in Solution Adopt Very Similar Ramachandran Map Distributions That Closely Resemble Random Coil. Biochemistry.

[13] Giulian D., Haverkamp L.J., Yu J., Karshin W., Tom D., Li J., Kazanskaia A., Kirkpatrick J., Roher A.E. (1998). The HHQK domain of beta-amyloid provides a structural basis for the immunopathology of Alzheimer’s disease. J. Biol. Chem..

[14] Tiiman A., Krishtal J., Palumaa P., Tõugu V. (2015). In vitro fibrillization of Alzheimer’s amyloid-b peptide (1-42). AIP Adv..

[15] Hilbich C., Kisters-Woike B., Reed J., Masters C.L., Beyreuther K. (1992). Substitutions of hydrophobic amino acids reduce the amyloidogenicity of Alzheimer’s disease beta A4 peptides. J. Mol. Biol..

[16] Serpell L.C. (2000). Alzheimer’s amyloid fibrils: Structure and assembly. Biochim. Biophys. Acta.

[17] Gazit E. (2002). A possible role for pi-stacking in the self-assembly of amyloid fibrils. FASEB J..

[18] de Groot N.S., Aviles F.X., Vendrell J., Ventura S. (2006). Mutagenesis of the central hydrophobic cluster in Abeta42 Alzheimer’s peptide. Side-chain properties correlate with aggregation propensities. FEBS J..

[19] Citron M. (2010). Alzheimer’s disease: Strategies for disease modification. Nat. Rev. Drug Discov..

[20] Warmlander S., Tiiman A., Abelein A., Luo J., Jarvet J., Soderberg K.L., Danielsson J., Graslund A. (2013). Biophysical studies of the amyloid beta-peptide: Interactions with metal ions and small molecules. Chembiochem.

[21] Kumar S., Henning-Knechtel A., Chehade I., Magzoub M., Hamilton A.D. (2017). Foldamer-Mediated Structural Rearrangement Attenuates Abeta Oligomerization and Cytotoxicity. J. Am. Chem. Soc..

[22] Kumar S., Henning-Knechtel A., Magzoub M., Hamilton A.D. (2018). Peptidomimetic-Based Multidomain Targeting Offers Critical Evaluation of Abeta Structure and Toxic Function. J. Am. Chem. Soc..

[23] Doody R.S., Raman R., Farlow M., Iwatsubo T., Vellas B., Joffe S., Kieburtz K., He F., Sun X., Thomas R.G. (2013). A phase 3 trial of semagacestat for treatment of Alzheimer’s disease. N. Engl. J. Med..

[24] Goyal D., Shuaib S., Mann S., Goyal B. (2017). Rationally Designed Peptides and Peptidomimetics as Inhibitors of Amyloid-beta (Abeta) Aggregation: Potential Therapeutics of Alzheimer’s Disease. ACS Comb. Sci..

[25] Deike S., Rothemund S., Voigt B., Samantray S., Strodel B., Binder W.H. (2020). beta-Turn mimetic synthetic peptides as amyloid-beta aggregation inhibitors. Bioorg. Chem..

[26] Kapadia A., Sharma K.K., Maurya I.K., Singh V., Khullar M., Jain R. (2021). Structural and mechanistic insights into the inhibition of amyloid-beta aggregation by Abeta(39-42) fragment derived synthetic peptides. Eur. J. Med. Chem..

[27] Kumar S., Srivastav S., Fatima M., Giri R.S., Mandal B., Mondal A.C. (2019). A Synthetic Pro-Drug Peptide Reverses Amyloid-beta-Induced Toxicity in the Rat Model of Alzheimer’s Disease. J. Alzheimer’s Dis..

[28] Shea D., Hsu C.C., Bi T.M., Paranjapye N., Childers M.C., Cochran J., Tomberlin C.P., Wang L., Paris D., Zonderman J. (2019). alpha-Sheet secondary structure in amyloid beta-peptide drives aggregation and toxicity in Alzheimer’s disease. Proc. Natl. Acad. Sci. USA.

[29] Baine M., Georgie D.S., Shiferraw E.Z., Nguyen T.P., Nogaj L.A., Moffet D.A. (2009). Inhibition of Abeta42 aggregation using peptides selected from combinatorial libraries. J. Pept. Sci..

[30] Rajasekhar K., Madhu C., Govindaraju T. (2016). Natural Tripeptide-Based Inhibitor of Multifaceted Amyloid beta Toxicity. ACS Chem. Neurosci..

[31] Henning-Knechtel A., Kuma S., Wallin C., Król S., Wärmländer S.K.T.S., Jarvet J., Esposito G., Kirmizialtin S., Gräslund A., Hamilton A.D. (2020). Designed Cell-Penetrating Peptide Inhibitors of Amyloid-beta Aggregation and Cytotoxicity. Cell Rep. Phys. Sci..

[32] Rajasekhar K., Suresh S.N., Manjithaya R., Govindaraju T. (2015). Rationally designed peptidomimetic modulators of abeta toxicity in Alzheimer’s disease. Sci. Rep..

[33] Konar M., Ghosh D., Samanta S., Govindaraju T. (2022). Combating amyloid-induced cellular toxicity and stiffness by designer peptidomimetics. RSC Chem. Biol..

[34] Bollati M., Peqini K., Barone L., Natale C., Beeg M., Gobbi M., Diomede L., Trucchi M., de Rosa M., Pellegrino S. (2022). Rational Design of a Peptidomimetic Inhibitor of Gelsolin Amyloid Aggregation. Int. J. Mol. Sci..

[35] Ramesh M., Govindaraju T. (2022). Multipronged diagnostic and therapeutic strategies for Alzheimer’s disease. Chem. Sci..

[36] De Lorenzi E., Chiari M., Colombo R., Cretich M., Sola L., Vanna R., Gagni P., Bisceglia F., Morasso C., Lin J.S. (2017). Evidence that the Human Innate Immune Peptide LL-37 may be a Binding Partner of Amyloid-beta and Inhibitor of Fibril Assembly. J. Alzheimer’s Dis..

[37] Iqbal U.H., Zeng E., Pasinetti G.M. (2020). The Use of Antimicrobial and Antiviral Drugs in Alzheimer’s Disease. Int. J. Mol. Sci..

[38] Armiento V., Hille K., Naltsas D., Lin J.S., Barron A.E., Kapurniotu A. (2020). The Human Host-Defense Peptide Cathelicidin LL-37 is a Nanomolar Inhibitor of Amyloid Self-Assembly of Islet Amyloid Polypeptide (IAPP). Angew. Chem. Int. Ed. Engl..

[39] Bruno F., Malvaso A., Canterini S., Bruni A.C. (2022). Antimicrobial Peptides (AMPs) in the Pathogenesis of Alzheimer’s Disease: Implications for Diagnosis and Treatment. Antibiotics.

[40] Cheng P.N., Liu C., Zhao M., Eisenberg D., Nowick J.S. (2012). Amyloid beta-sheet mimics that antagonize protein aggregation and reduce amyloid toxicity. Nat. Chem..

[41] Seither K.M., McMahon H.A., Singh N., Wang H., Cushman-Nick M., Montalvo G.L., DeGrado W.F., Shorter J. (2014). Specific aromatic foldamers potently inhibit spontaneous and seeded Abeta42 and Abeta43 fibril assembly. Biochem. J..

[42] Kumar S., Birol M., Miranker A.D. (2016). Foldamer scaffolds suggest distinct structures are associated with alternative gains-of-function in a preamyloid toxin. Chem. Commun..

[43] Zhu L., Song Y., Cheng P.N., Moore J.S. (2015). Molecular Design for Dual Modulation Effect of Amyloid Protein Aggregation. J. Am. Chem. Soc..

[44] Tjernberg L.O., Naslund J., Lindqvist F., Johansson J., Karlstrom A.R., Thyberg J., Terenius L., Nordstedt C. (1996). Arrest of beta-amyloid fibril formation by a pentapeptide ligand. J. Biol. Chem..

[45] Tjernberg L.O., Lilliehook C., Callaway D.J., Naslund J., Hahne S., Thyberg J., Terenius L., Nordstedt C. (1997). Controlling amyloid beta-peptide fibril formation with protease-stable ligands. J. Biol. Chem..

[46] Horsley J.R., Jovcevski B., Wegener K.L., Yu J., Pukala T.L., Abell A.D. (2020). Rationally designed peptide-based inhibitor of Abeta42 fibril formation and toxicity: A potential therapeutic strategy for Alzheimer’s disease. Biochem. J..

[47] Soto C., Kindy M.S., Baumann M., Frangione B. (1996). Inhibition of Alzheimer’s amyloidosis by peptides that prevent beta-sheet conformation. Biochem. Biophys. Res. Commun..

[48] Minicozzi V., Chiaraluce R., Consalvi V., Giordano C., Narcisi C., Punzi P., Rossi G.C., Morante S. (2014). Computational and experimental studies on beta-sheet breakers targeting Abeta1-40 fibrils. J. Biol. Chem..

[49] Datki Z., Papp R., Zadori D., Soos K., Fulop L., Juhasz A., Laskay G., Hetenyi C., Mihalik E., Zarandi M. (2004). In vitro model of neurotoxicity of Abeta 1-42 and neuroprotection by a pentapeptide: Irreversible events during the first hour. Neurobiol. Dis..

[50] Jagota S., Rajadas J. (2013). Synthesis of D-amino acid peptides and their effect on beta-amyloid aggregation and toxicity in transgenic Caenorhabditis elegans. Med. Chem. Res..

[51] Gilead S., Gazit E. (2004). Inhibition of amyloid fibril formation by peptide analogues modified with alpha-aminoisobutyric acid. Angew. Chem. Int. Ed. Engl..

[52] Mishra A., Misra A., Vaishnavi T.S., Thota C., Gupta M., Ramakumar S., Chauhan V.S. (2013). Conformationally restricted short peptides inhibit human islet amyloid polypeptide (hIAPP) fibrillization. Chem. Commun..

[53] Loureiro J.A., Crespo R., Borner H., Martins P.M., Rocha F.A., Coelho M., Pereira M.C., Rocha S. (2014). Fluorinated beta-sheet breaker peptides. J. Mater. Chem. B.

[54] Sinopoli A., Giuffrida A., Tomasello M.F., Giuffrida M.L., Leone M., Attanasio F., Caraci F., De Bona P., Naletova I., Saviano M. (2016). Ac-LPFFD-Th: A Trehalose-Conjugated Peptidomimetic as a Strong Suppressor of Amyloid-beta Oligomer Formation and Cytotoxicity. Chembiochem.

[55] Findeis M.A., Lee J.J., Kelley M., Wakefield J.D., Zhang M.H., Chin J., Kubasek W., Molineaux S.M. (2001). Characterization of cholyl-leu-val-phe-phe-ala-OH as an inhibitor of amyloid beta-peptide polymerization. Amyloid.

[56] Wei C.W., Peng Y., Zhang L., Huang Q., Cheng M., Liu Y.N., Li J. (2011). Synthesis and evaluation of ferrocenoyl pentapeptide (Fc-KLVFF) as an inhibitor of Alzheimer’s Abeta(1)-(4)(2) fibril formation in vitro. Bioorg. Med. Chem. Lett..

[57] Arai T., Araya T., Sasaki D., Taniguchi A., Sato T., Sohma Y., Kanai M. (2014). Rational design and identification of a non-peptidic aggregation inhibitor of amyloid-beta based on a pharmacophore motif obtained from cyclo[-Lys-Leu-Val-Phe-Phe-]. Angew. Chem. Int. Ed. Engl..

[58] Ikenoue T., Aprile F.A., Sormanni P., Ruggeri F.S., Perni M., Heller G.T., Haas C.P., Middel C., Limbocker R., Mannini B. (2020). A rationally designed bicyclic peptide remodels Abeta42 aggregation in vitro and reduces its toxicity in a worm model of Alzheimer’s disease. Sci. Rep..

[59] Puneeth Kumar D.R., Reja R.M., Senapati D.K., Singh M., Nalawade S.A., George G., Kaul G., Akhir A., Chopra S., Raghothama S. (2023). A cationic amphiphilic peptide chaperone rescues Abeta(42) aggregation and cytotoxicity. RSC Med. Chem..

[60] Puneeth Kumar D.R., Nalawade S.A., Pahan S., Singh M., Senapati D.K., Roy S., Dey S., Toraskar S.U., Raghothama S., Gopi H.N. (2023). Proteolytically Stable alphaalphagamma-Hybrid Peptides Inhibit the Aggregation and Cytotoxicity of Abeta(42). ACS Chem. Neurosci..

[61] Pariary R., Shome G., Kalita S., Kalita S., Roy A., Harikishore A., Jana K., Senapati D., Mandal B., Mandal A.K. (2024). Peptide-Based Strategies: Combating Alzheimer’s Amyloid beta Aggregation through Ergonomic Design and Fibril Disruption. Biochemistry.

[62] Bodenhausen G., Ruben D.J. (1980). Natural abundance nitrogen-15 NMR by enhanced heteronuclear spectroscopy. Chem. Phys. Lett..

[63] Khandogin J., Brooks C.L. (2007). Linking folding with aggregation in Alzheimer’s beta-amyloid peptides. Proc. Natl. Acad. Sci. USA.

[64] Thirumalai D., Reddy G., Straub J.E. (2012). Role of water in protein aggregation and amyloid polymorphism. Acc. Chem. Res..

[65] Xue C., Lin T.Y., Chang D., Guo Z. (2017). Thioflavin T as an amyloid dye: Fibril quantification, optimal concentration and effect on aggregation. R. Soc. Open Sci..

[66] Vadukul D.M., Gbajumo O., Marshall K.E., Serpell L.C. (2017). Amyloidogenicity and toxicity of the reverse and scrambled variants of amyloid-beta 1-42. FEBS Lett..

[67] Keskitalo S., Farkas M., Hanenberg M., Szodorai A., Kulic L., Semmler A., Weller M., Nitsch R.M., Linnebank M. (2014). Reciprocal modulation of Abeta42 aggregation by copper and homocysteine. Front. Aging Neurosci..

[68] Ultsch M., Li B., Maurer T., Mathieu M., Adolfsson O., Muhs A., Pfeifer A., Pihlgren M., Bainbridge T.W., Reichelt M. (2016). Structure of Crenezumab Complex with Abeta Shows Loss of beta-Hairpin. Sci. Rep..

[69] Fawzi N.L., Ying J., Ghirlando R., Torchia D.A., Clore G.M. (2011). Atomic-resolution dynamics on the surface of amyloid-beta protofibrils probed by solution NMR. Nature.

[70] Hong S., Baravkar S.B., Lu Y., Masoud A.R., Zhao Q., Zhou W. (2025). Molecular Modification of Queen Bee Acid and 10-Hydroxydecanoic Acid with Specific Tripeptides: Rational Design, Organic Synthesis, and Assessment for Prohealing and Antimicrobial Hydrogel Properties. Molecules.

[71] Baravkar S.B., Lu Y., Masoud A.R., Zhao Q., He J., Hong S. (2024). Development of a Novel Covalently Bonded Conjugate of Caprylic Acid Tripeptide (Isoleucine-Leucine-Aspartic Acid) for Wound-Compatible and Injectable Hydrogel to Accelerate Healing. Biomolecules.

[72] Hong S., Baravkar S.B., Lu Y. (2024). Amphiphilic Conjugates of Fatty Acids or their Derivatives. US Provisional Patent.

[73] Oakley H., Cole S.L., Logan S., Maus E., Shao P., Craft J., Guillozet-Bongaarts A., Ohno M., Disterhoft J., Van Eldik L. (2006). Intraneuronal beta-amyloid aggregates, neurodegeneration, and neuron loss in transgenic mice with five familial Alzheimer’s disease mutations: Potential factors in amyloid plaque formation. J. Neurosci..

[74] Drummond E., Wisniewski T. (2017). Alzheimer’s disease: Experimental models and reality. Acta Neuropathol..

[75] Myers A., McGonigle P. (2019). Overview of Transgenic Mouse Models for Alzheimer’s Disease. Curr. Protoc. Neurosci..

[76] Jacob H.J., Kwitek A.E. (2002). Rat genetics: Attaching physiology and pharmacology to the genome. Nat. Rev. Genet..

[77] Cohen R.M., Rezai-Zadeh K., Weitz T.M., Rentsendorj A., Gate D., Spivak I., Bholat Y., Vasilevko V., Glabe C.G., Breunig J.J. (2013). A transgenic Alzheimer rat with plaques, tau pathology, behavioral impairment, oligomeric abeta, and frank neuronal loss. J. Neurosci..

[78] Do Carmo S., Cuello A.C. (2013). Modeling Alzheimer’s disease in transgenic rats. Mol. Neurodegener..

[79] Berkowitz L.E., Harvey R.E., Drake E., Thompson S.M., Clark B.J. (2018). Progressive impairment of directional and spatially precise trajectories by TgF344-Alzheimer’s disease rats in the Morris Water Task. Sci. Rep..

[80] Lochhead J.J., Thorne R.G. (2012). Intranasal delivery of biologics to the central nervous system. Adv. Drug Deliv. Rev..

[81] Chauhan M.B., Chauhan N.B. (2015). Brain Uptake of Neurotherapeutics after Intranasal versus Intraperitoneal Delivery in Mice. J. Neurol. Neurosurg..

[82] Erdo F., Bors L.A., Farkas D., Bajza A., Gizurarson S. (2018). Evaluation of intranasal delivery route of drug administration for brain targeting. Brain Res. Bull..

