# Supplementary material for: Rationally Designed Pentapeptide Analogs of Aβ19–23 Fragment as Potent Inhibitors of Aβ42 Aggregation
Source: Molecules. 2025 May 7;30(9):2071. doi: 10.3390/molecules30092071 (PMC12073614; doi:10.3390/molecules30092071)
Supplement: Supplementary file 1 [file molecules-30-02071-s001.zip › molecules-3494009-supplementary.pdf]

Rationally Designed Pentapeptide Analogs of Aβ19–23 Fragment as Potent Inhibitors of Aβ42-Aggregation

Sachin B. Baravkar[1,#], Yan Lu[1,#], Qi Zhao[2], Hongying Peng[3], Weilie Zhou[4], Song Hong[1,5,*]

1 Neuroscience Center of Excellence, School of Medicine, L.S.U. Health, New Orleans, LA 70112, U.S.A.
2 NMR Laboratory, Department of Chemistry, Tulane University, New Orleans, LA 70115, U.S.A.
3 Department of Environmental Health, University of Cincinnati College of Medicine, Cincinnati, OH, 45221, USA
4 Department of Physics & AMRI, University of New Orleans, LA 70148, U.S.A.
5 Department of Ophthalmology, School of Medicine, L.S.U. Health, New Orleans, LA 70112, U.S.A.
* Correspondence: Song Hong, PhD, shong@lsuhsc.edu
\# Share first authorship

NMR Spectra

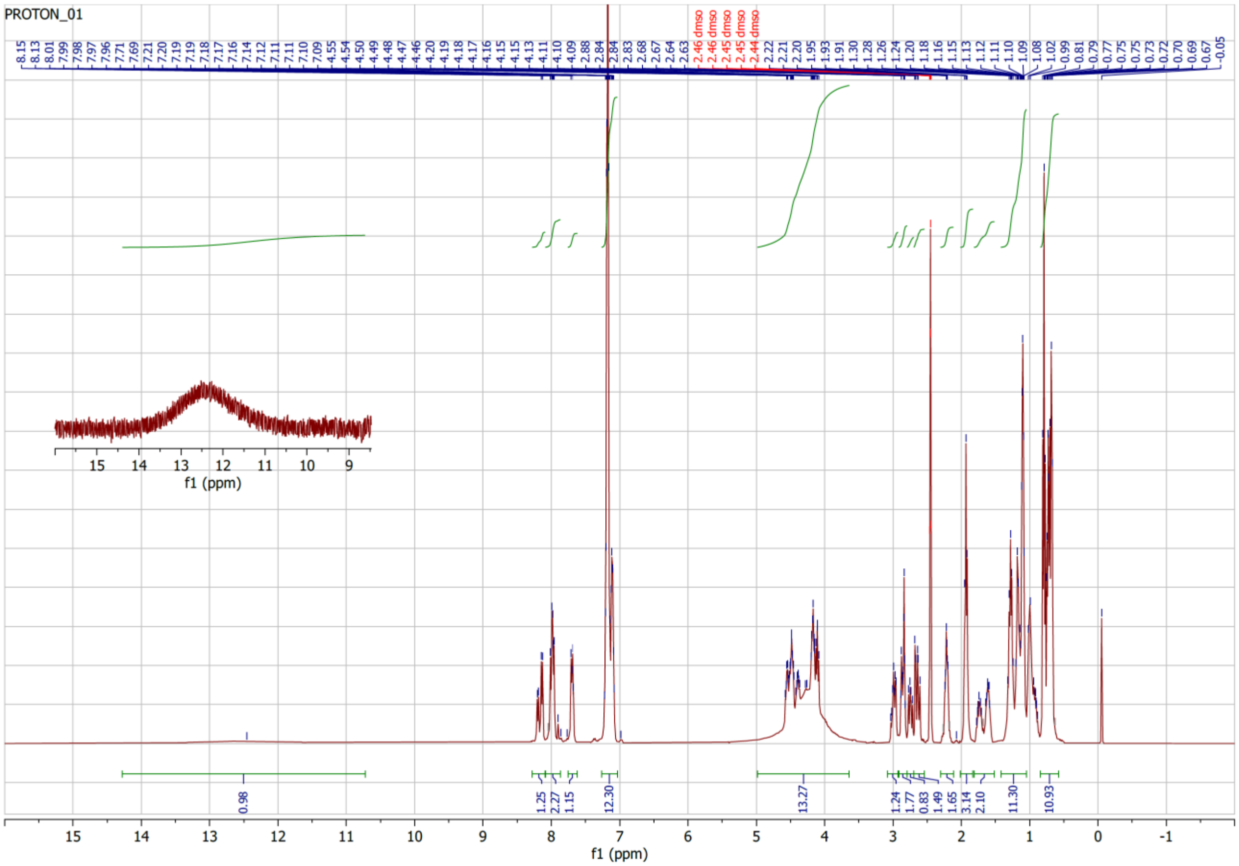

Fig. S 1: ^{1}H NMR spectrum of peptide **1** (DMSO-d6)

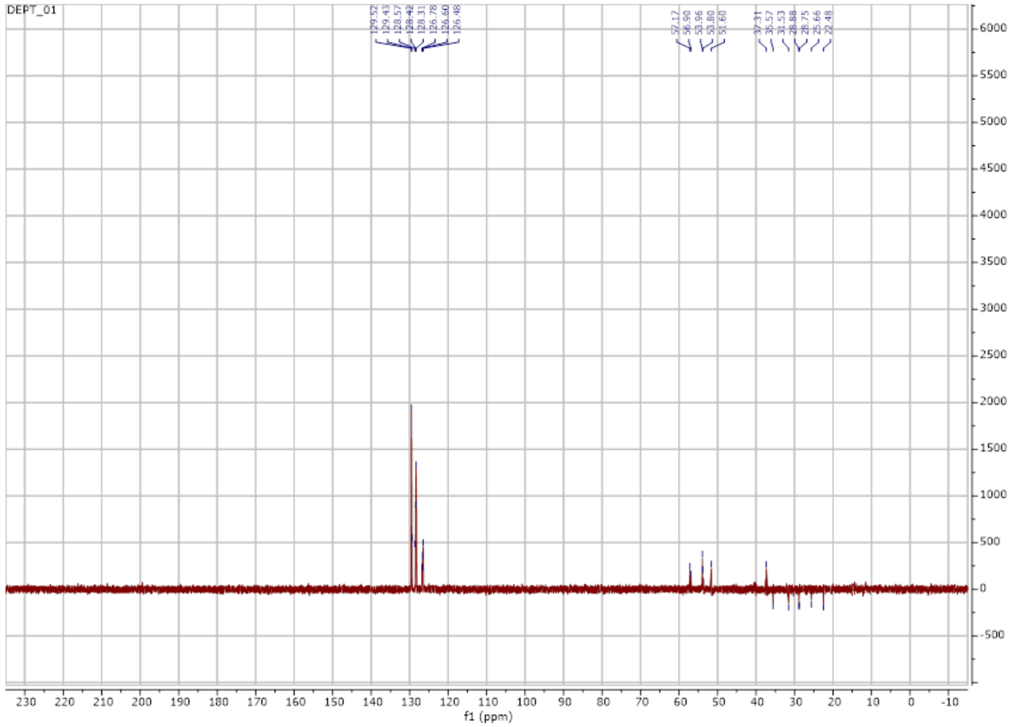

Fig. S2: ^{13}C DEPT NMR spectrum of peptide **1** (DMSO-d6)

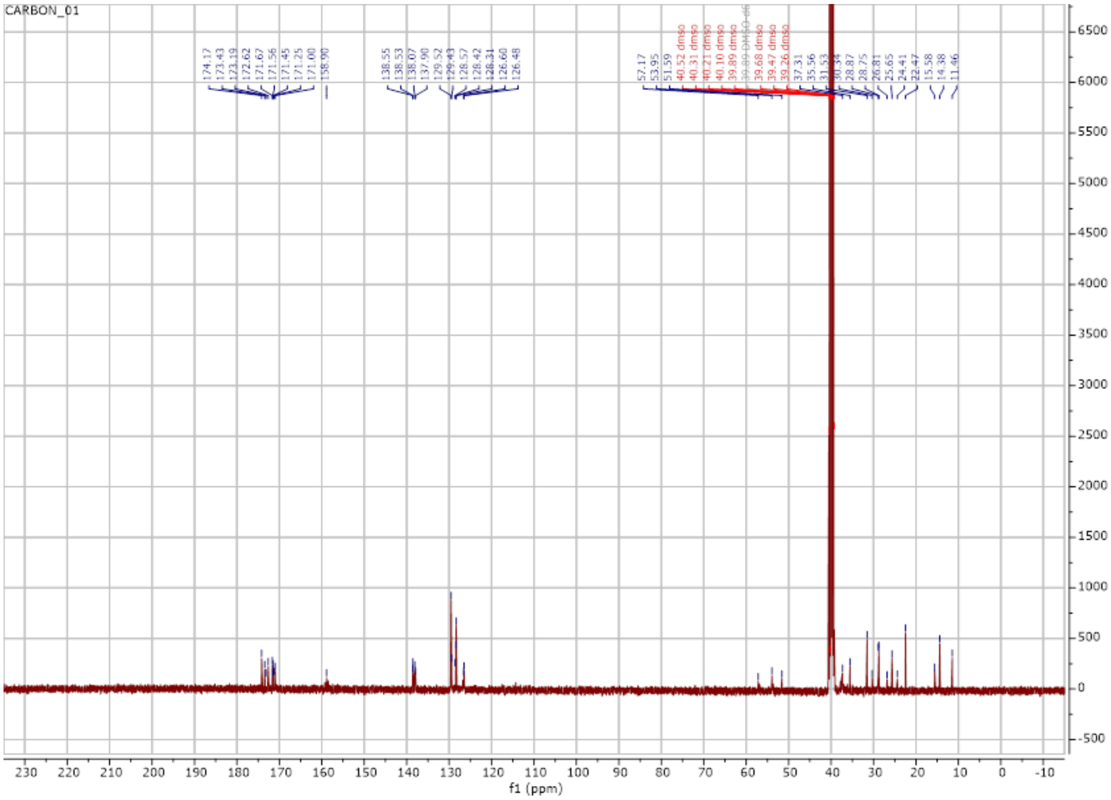

Fig. S3: ^{13}C NMR spectrum of peptide **1** (DMSO-d6)

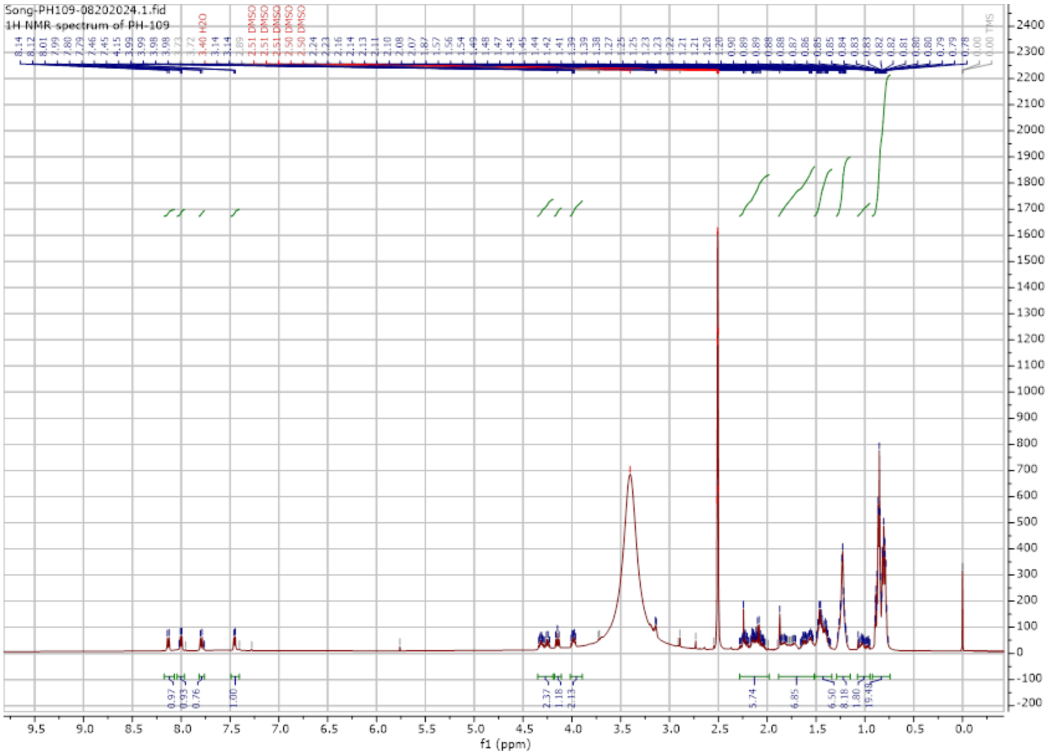

Fig. S4: ^{1}H NMR spectrum of peptide **2** (DMSO-d6)

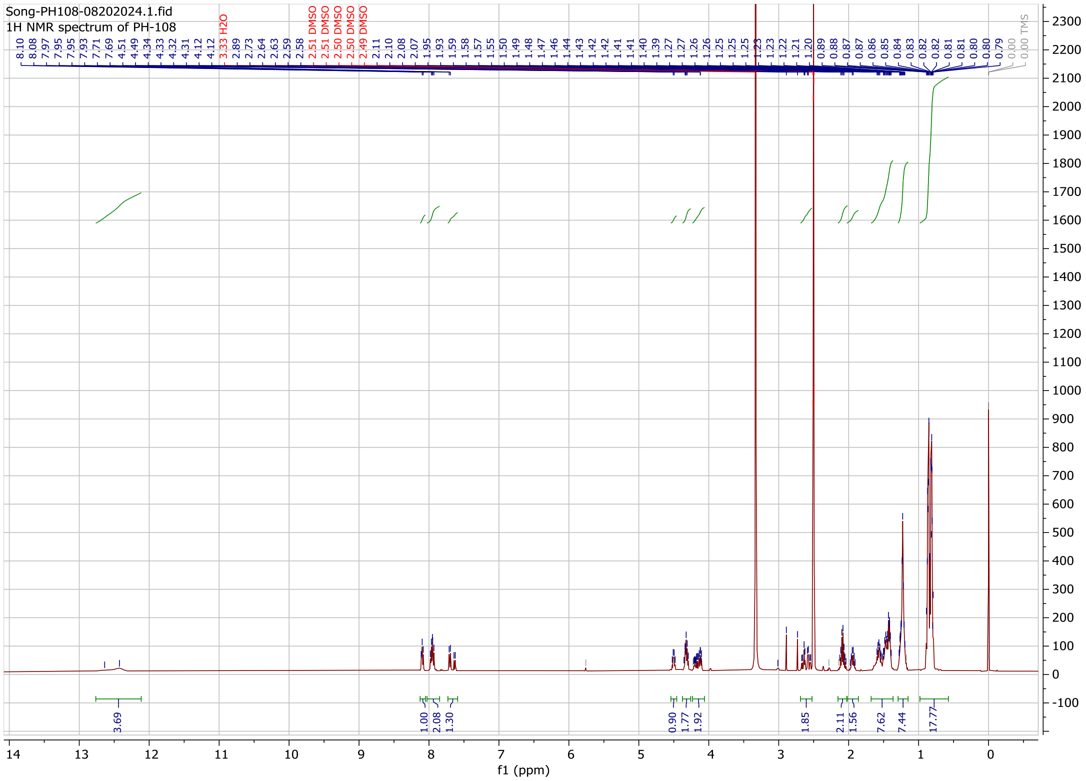

Fig. S5: ^{1}H NMR spectrum of peptide **3** (DMSO-d6)

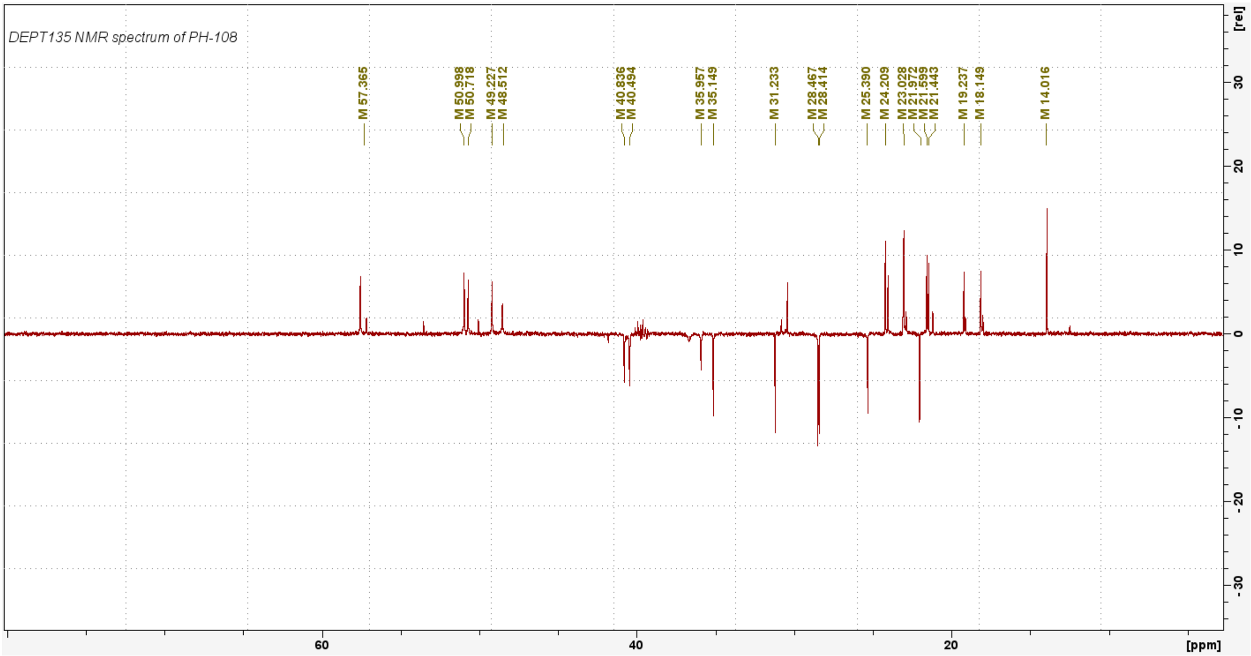

Fig. S6: ^{13}C DEPT NMR spectrum of peptide **3** (DMSO-d6)

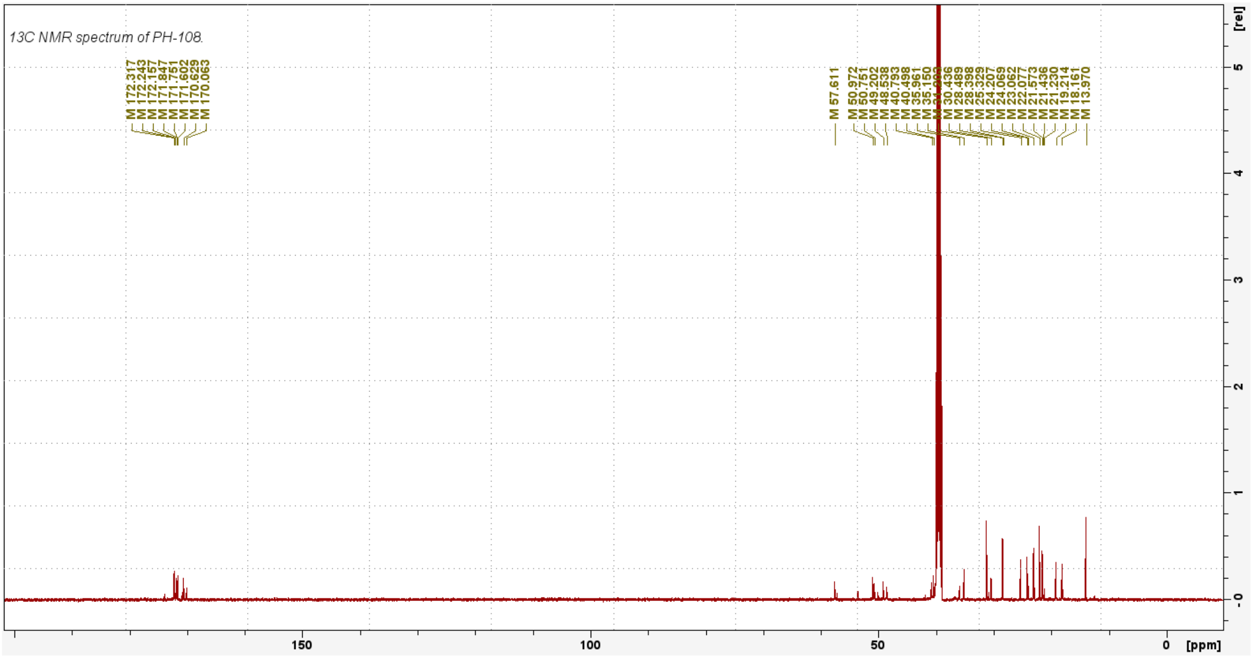

Fig. S7. 13C NMR spectrum of peptide **3** (DMSO-d6)

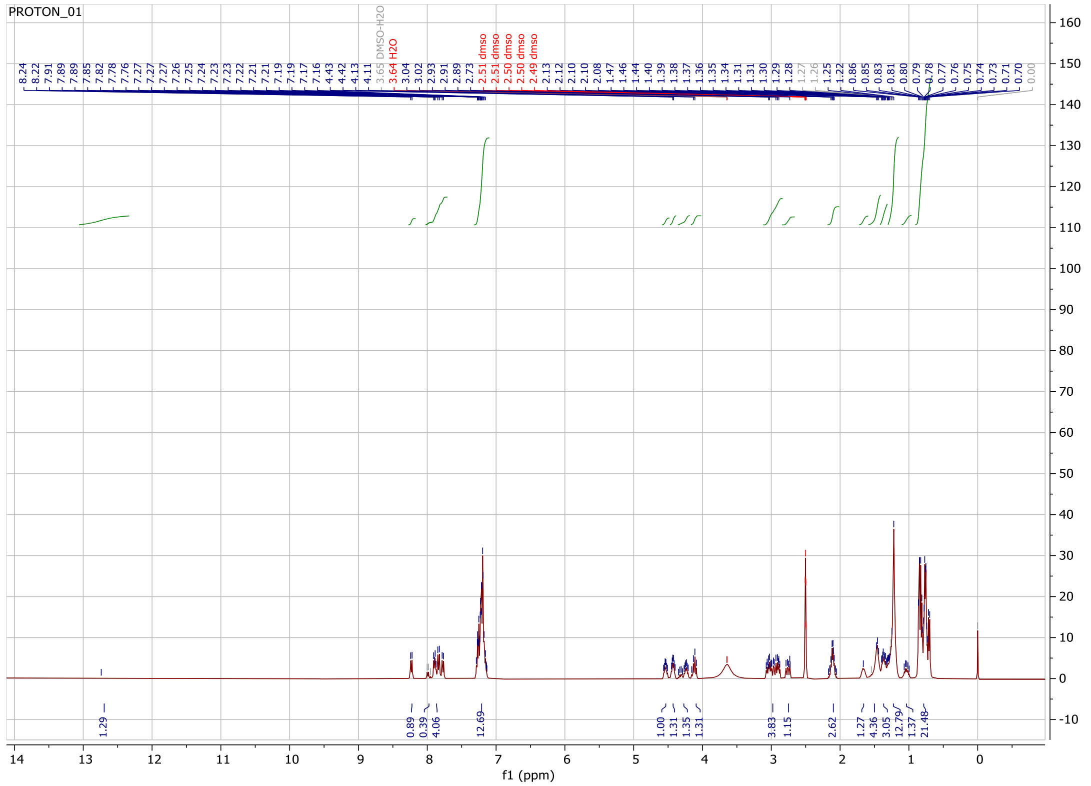

Fig. S8: ¹H NMR spectrum of peptide **4** (DMSO-d6)

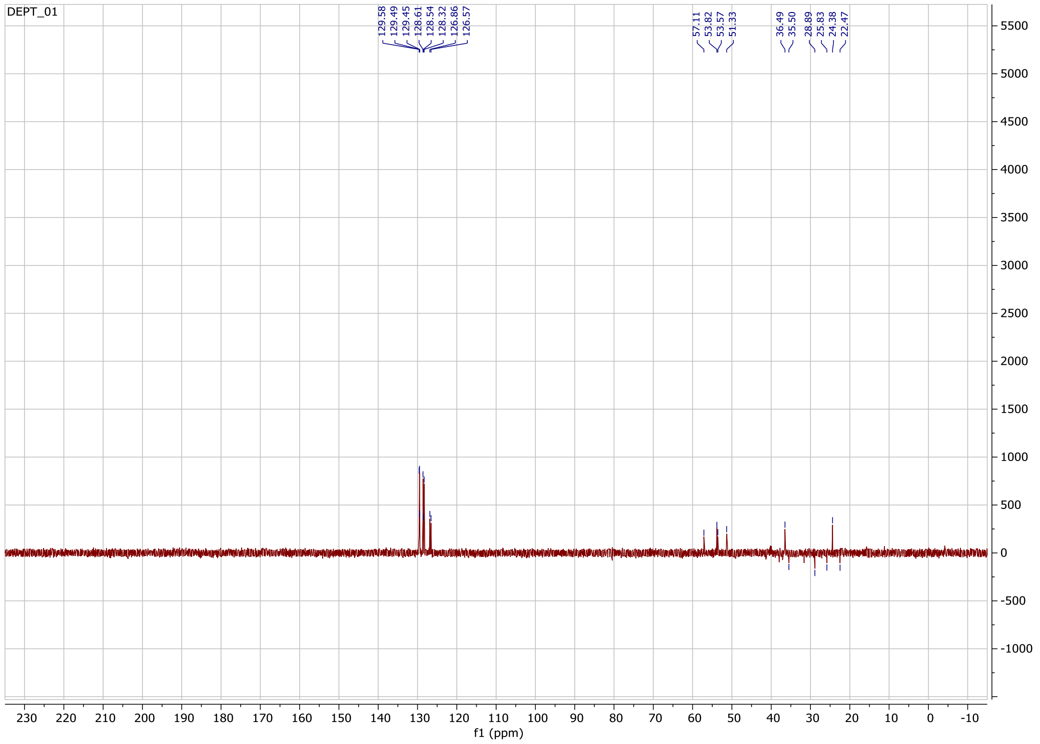

Fig. S9. 13C DEPT NMR spectrum of peptide **4** (DMSO-d6)

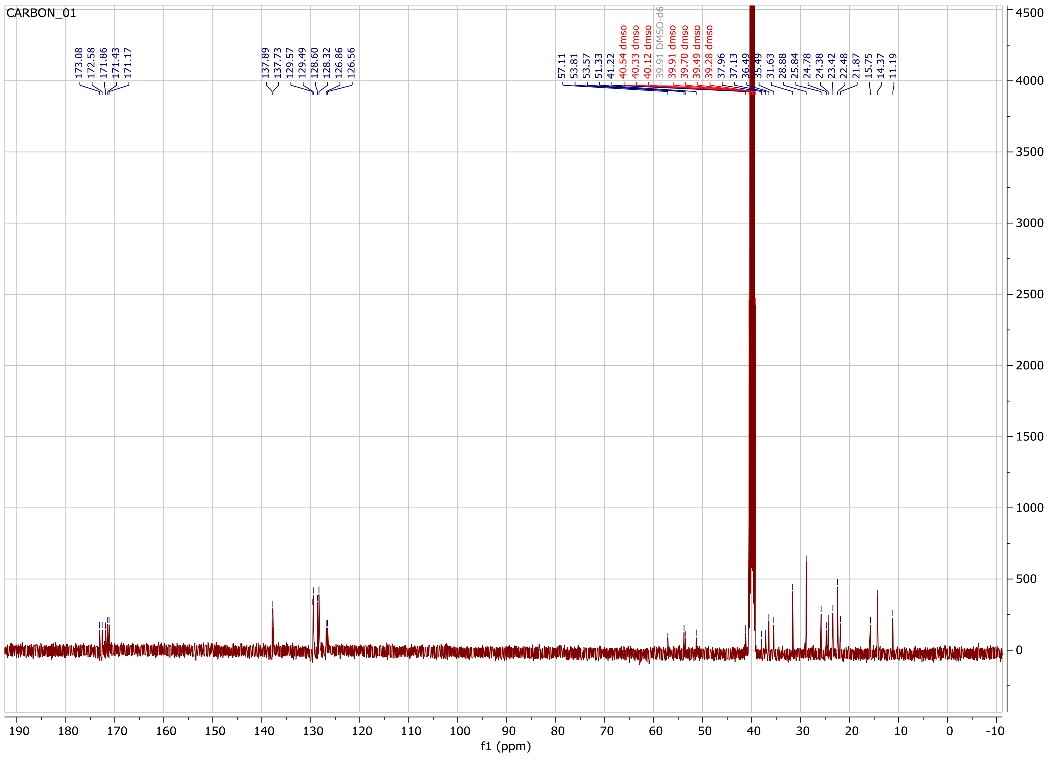

Fig. S10: ^{13}C NMR of peptide **4** (DMSO-d6)

**Amino Acid Residue**	**Δppm, ^{15}N**
E3	0.01
F4	-0.06
R5	-0.02
D7	-0.02
S8	-0.03
G9	0.10
Y10	0.01
E11	-0.02
V12	-0.02
H13	-0.01
Q15	0.01
K16	-0.09
L17	0.04
V18	0.02
F19	0.08
F20	-0.04
A21	0.01
E22	-0.03
D23	0.01
V24	0.00
G25	0.03
S26	0.01
N27	0.03
K28	0.02
G29	-0.02
A30	0.04
I31	0.01
I32	0.01
G33	0.00
L34	-0.01
M35	0.01
V36	-0.02
G37	-0.07
G38	0.06
V39	0.02
V40	-0.01
I41	0.06

Table S1. ^{15}N chemical shifts of ^{15}N-labeled Aβ42 with and without inhibitor peptide 3 in the ^{1}H–^{15}N HSQC NMR experiments.

Amino Acid Residue	^{15}N-labeled Aβ42, Peak Intensity	^{15}N-labeled Aβ42 + peptide 3, Peak Intensity
E3	1.54E+09	1.47E+09
F4	1.39E+09	1.48E+09
R5	1.00E+09	8.94E+08
D7	1.49E+09	1.37E+09
S8	7.43E+08	5.43E+08
G9	5.93E+08	4.80E+08
Y10	1.25E+09	9.96E+08
E11	1.05E+09	9.55E+08
V12	1.35E+09	9.23E+08
H13	6.16E+08	6.68E+08
Q15	7.09E+08	6.29E+08
K16	7.98E+08	5.81E+08
L17	9.51E+08	8.20E+08
V18	1.10E+09	1.01E+08
F19	8.90E+08	8.97E+08
F20	1.20E+09	1.72E+09
A21	1.09E+09	1.05E+09
E22	2.01E+09	1.63E+09
D23	2.31E+09	2.24E+09
V24	2.59E+09	1.34E+09
G25	1.59E+09	1.38E+09
S26	1.02E+09	1.09E+09
N27	1.55E+09	1.47E+09
K28	1.49E+09	1.37E+09
G29	1.29E+09	1.27E+09
A30	2.06E+09	1.99E+09
I31	2.59E+09	1.21E+09
I32	1.60E+09	1.56E+09
G33	1.25E+09	1.12E+09
L34	2.35E+09	2.45E+09
M35	2.31E+09	2.24E+09
V36	1.94E+09	1.74E+09
G37	7.93E+08	6.58E+08
G38	1.23E+09	1.17E+09
V39	2.23E+09	1.85E+09
V40	2.46E+09	2.55E+09
I41	2.36E+09	2.52E+09
A42		8.35E+08

Table S2. Peak intensities of ^{15}N-labeled Aβ42 with and without inhibitor peptide 3 in the ^{1}H–^{15}N HSQC NMR experiments.
